# P12 National variation in antimicrobial management of ‘sepsis of unknown origin’

**DOI:** 10.1093/jacamr/dlaf118.019

**Published:** 2025-07-14

**Authors:** Isobel P Soper, Matthew Youngman, Nick K Jones

**Affiliations:** Psychiatry, Norfolk & Suffolk Foundation Trust, UK; Department of Pharmacy, West Suffolk Hospital, Bury St Edmunds, UK; Microbiology Department, West Suffolk Hospital, Bury St Edmunds, UK

## Abstract

**Background:**

Antimicrobial treatment is a cornerstone of sepsis management. When the underlying infection source is unknown, empirical regimens aim to provide broad-spectrum antimicrobial activity, while minimizing off-target effects. In the absence of UK national guidance, antimicrobial selection for adult patients with sepsis of unknown origin (SUO) is determined by local hospital practice. National variation in SUO recommendations is neither routinely monitored, nor well characterized. We sought to investigate the degree of variation in local antimicrobial guidelines for severe community-onset SUO and describe the antimicrobial stewardship impact of penicillin allergy in SUO.

**Methods:**

The Eolas clinical guidelines platform was searched for all subscribing NHS Trusts during January–March 2025. Only Trusts providing emergency care to unselected patient groups were included. Access to guidelines for the explicit purpose of the project was requested from Trusts with access restrictions in place. Guidelines were systematically reviewed for treatment recommendations for adult patients with severe community-onset ‘sepsis of unknown origin’. Where guidelines were stratified by degrees of severity, recommendations from the most severe category were preferentially included. If distinct antimicrobial recommendations were listed for different hospital sites from the same Trust, each antimicrobial regimen was included separately. Optional adjunctive agents were omitted from the analysis unless haemodynamic instability or severe/life-threatening sepsis were listed as indications for their inclusion. The guideline search assumed treatment of a patient aged 18–65 years, with creatinine clearance >20 mL/min and body weight 65 kg.

**Results:**

Of 109 NHS Trusts on the Eolas app meeting inclusion criteria, 45 had openly accessible adult antimicrobial guidelines. A further 34 granted guideline access for inclusion in the project. 78/79 (99%) Trusts had specific guidelines for SUO. 73/78 (94%) provided separate recommendations for penicillin allergic patients, with 72/78 (92%) differentiating ‘mild-moderate’ allergy from ‘severe’. 22 distinct antimicrobial regimens were recommended for non-allergic patients, compared with 31 for mild-moderate penicillin allergy and 25 for undifferentiated or severe allergy. The most frequently recommended regimens were co-amoxiclav + gentamicin for non-allergic patients (18/78; 23%), meropenem for mild-moderate penicillin allergy (10/72; 14%), and gentamicin + teicoplanin + metronidazole for undifferentiated/severe penicillin allergy (14/73; 19%). Non-penicillin β-lactams were frequently recommended for patients with mild-moderate penicillin allergy; cephalosporins in 27/72 (38%), meropenem in 12/72 (17%), and aztreonam in 1/72 (1%). β-Lactams also featured in guidelines for undifferentiated/severe penicillin allergy; cephalosporins in 1/73 (1%), meropenem in 1/73 (1%), and aztreonam in 3/73 (4%). Use of antimicrobial agents from the WHO ‘Watch’ and ‘Reserve’ groups was avoided entirely in 21/78 (27%) guidelines for non-allergic patients, compared to only 4/72 for mild-moderate penicillin allergy (6%) and 3/73 (4%) for undifferentiated/severe penicillin allergy. 61/78 (78%) guidelines for non-allergic patients included an aminoglycoside, with amikacin preferred to gentamicin in 7/61 (12%). Recommended gentamicin dosing was 5 mg/kg in 46/54 (85%) guidelines and 7mg/kg in 58/54 (15%). 6/23 (26%) guidelines that included piperacillin-tazobactam recommended QDS dosing.

**Conclusions:**

Antimicrobial guidelines for SUO are highly heterogenous in the UK, with notable variation in antimicrobial selection and dosing. Penicillin allergy is a barrier to the avoidance of ‘Watch’ and ‘Reserve’ group antimicrobials.

